# Transungual Delivery, Anti-Inflammatory Activity, and In Vivo Assessment of a Cyclodextrin Polypseudorotaxanes Nail Lacquer

**DOI:** 10.3390/pharmaceutics12080730

**Published:** 2020-08-04

**Authors:** Francisco Fernández-Campos, Francesc Navarro, Adrian Corrales, Jordi Picas, Eloy Pena, Jordi González, Francisco J. Otero-Espinar

**Affiliations:** 1 R & D Department. Reig Jofre Laboratories, Avda dels Flors, s/n, 08970 Sant Joan Despi, Spain; fnavarro@reigjofre.com (F.N.); acorrales@reigjofre.com (A.C.); jpicas@reigjofre.com (J.P.); epena@reigjofre.com (E.P.); jgplagaro@gmail.com (J.G.); 2 Department of Pharmacology, Pharmacy and Pharmaceutical Technology, Faculty of Pharmacy, University of Santiago de Compostela (USC), 15782 Santiago de Compostela, Spain; francisco.otero@usc.es; 3 Paraquasil Group, Health Research Institute of Santiago de Compostela (FIDIS), 15782 Santiago de Compostela, Spain

**Keywords:** nail lacquers, cyclodextrins, methylsulfonylmethane, active penetration, biotin

## Abstract

A new cyclodextrin polypseudorotaxanes nail lacquer (Regenail^®^) containing biotin, methyl sulphonyl methane (MSM), and dimethylsilanediol salicylate was developed and evaluated in vitro and in vivo. The product was developed to improve nail status and diminish signs of pathological nail alterations. A reference product (Betalfatrus^®^) was used for comparative purposes. An in vitro permeation experiment in hooves showed high MSM and biotin absorption. The content of sulfur and silicon in hooves was also found to be higher compared with the reference product. MSM was tested in human keratinocytes, exhibiting a good cytotoxicity profile and anti-inflammatory activity by the reduction in IL-8 and TNF-α under LPS stimuli. A clinical study was performed to check product safety and efficacy against nail brittleness and alterations such as Beau’s lines and onychorrhexis. A reduction in both alterations and in surface roughness without alteration of nail structure was observed, with a good level of patient acceptance and satisfaction.

## 1. Introduction

Nails are a complex structure composed of a nail bed (the deeper layer), nail matrix, nail folds, and the outer layer, nail plate. The nail bed is formed of a noncornified living epithelium tissue underlying the papilla dermis and contains blood vessels, nerves, and lymphatic fluid [1]. The nail plate contains between 80 and 90 layers of dense keratinized (mainly α-keratin) dead cells, with desmosome unions. This structure protects the nail bed from external damage. Nail folds are attached to the nail matrix and are a continuation of the skin, developing the cuticle, which seals the nail surface, preventing the entrance of chemicals and microorganisms. Finally, the nail plate is the outermost layer, formed by translucent dead keratinocytes and with a high keratin content, rich in cysteine, glycine, and tyrosine proteins. Lunula is the most characteristic distal semicircular white section of the nail plate [2,3,4]. Unlike the skin, nails have a low lipid content (around 1%), mainly composed of cholesterol, and the water content is higher than in the stratum corneum at around 10–30% (as a protein solvate) in a normal state [4]. These facts mean nails have different permeability properties from skin, which should be considered in formula development.

Different factors could affect nail status, such as environmental conditions, external factors, and several diseases. Environmental factors (e.g., low relative humidity, winter, etc.) could reduce the water content, leading to nail brittleness. Constant use of nail cosmetic products (nail polish) based on a high amount of organic solvents (e.g., butyl acetate, ethyl acetate) could extract nail water and lipids, leading to progressive dehydration. These products also contain chemical compounds (plasticizers, i.e., dibutyl phthalate and dioctyl phthalate; pearlizers, i.e., bismuth oxychloride; resins, i.e., toluene sulphonamide formaldehyde, shellac; etc.), that could induce contact dermatitis and nail plate coloration [5]. Nutritional deficiencies (biotin deficiencies [6]) and diseases (i.e., microbial infections, psoriasis, lichen planus, alopecia areata, Darier’s disease, etc.) could affect the nail structure [7,8].

Fungal infections (onychomycosis) are one of the most common nail diseases and are highly recurrent [9,10]. The prevalence ranged between 8% and 14% in North America, increasing with age up to 50% in the elderly [11,12]. They could be caused by dermatophytes, with *Trichophyton rubrum* being the main microorganism involved in around 71% of fungal infections, and the second most common cause being *Trichophyton mentagrophyte* (20%). Nondermatophyte microorganisms are less common, with *Candida albicans* responsible for 5.6% of nail infections [12]. Several clinical presentations could be present—hyperkeratosis, onycholysis, dyschromia (melanonychia), longitudinal striates, and inflammation—leading to nail structure alteration and function impairment. Topical antifungals are usually employed, and an oral alternative is reserved to treat more invasive and/or extensive manifestations [11].

Onychomycosis is present in 18% of patients, with nail psoriasis being an aggravating factor of the disease [13]. Psoriasis is a chronic immune disease affecting the skin. Keratinocyte hyperproliferation and inflammation are observed in patients and the involvement of the nail apparatus happens in around 50% of cases [14]. Nail alterations in psoriasis usually involve the nail matrix (pitting, leukonychia or white spots, and crumbling) and the nail bed (onycholysis, hyperkeratosis, and discoloration). In addition, longitudinal (onychorrhexis) and transversal ridges (also known as Beau’s lines) are usually present as a clinical manifestation.

Topical drug delivery systems to treat nail alterations are usually preferred to avoid the adverse effects of systemic medications. In addition, they have good patient acceptance and are cost-effective. The main drawback of topical therapy is low drug absorption due to the limited permeability of the nail structure, reducing the access of the drug to the nail bed. In addition, the nail bed turnover increases the reduction in drug concentrations [15]. To maintain an effective drug flux, several strategies (physical and chemical) are reported in the literature. Physical agents such as iontophoresis have been reported to increase antifungal and corticoid concentrations in the nail structure, improving therapeutic outcomes. Ultrasounds, photodynamic and laser therapy were also described as promising tools to increase drug effectiveness [12]. Physical abrasion with sandpaper aimed to reduce nail thickness and thereby increase drug diffusion across the ungual structure. This has been reported to increase terbinafine effectiveness in onychomycosis [16].

Transient chemical modification of the nail structure by permeation enhancers to increase drug diffusion is widely described in the literature. Considering that the main constituent of the nail is keratin, an alteration in protein packing is one mechanism of enhancement. Sulfhydryl compounds, such as cysteine, acetyl cysteine, and mercaptoethanol [17], and papaine, an endopeptidase with reactive sulfhydryl residues, reduce disulfide bounds and destabilize keratin. Softening compounds (urea and salicylic acid) have keratolytic properties and increase water uptake and swell keratin, making it less dense and compact and increasing the pore size [4]. Surfactants such as sodium lauryl sulfate could also alter the protein packing by electrostatic repulsion, induce micelle formation and absorption promotion, and reduce the contact angle between the formulation and nail surface to ensure better water access, increasing swelling [18]. Cyclodextrins are cyclic oligosaccharides and had the ability to form complexes with various active ingredients, involving the active ingredient in the lipophilic center. Their complex formation allows us to change the physical and chemical properties of the active ingredients [19,20]. Cyclodextrins had been reported to increase drug absorption across nails due to the solubilization of hydrophobic molecules—increasing the hydration of the nail plate, making pores broaden, and interacting with aromatic amino acids, limiting the protein folding. It was also demonstrated that the water itself could act as an ungual enhancer by the hydration and swelling of keratin fibers [21,22,23]. In addition, the hydroalcoholic-based formulation causes a higher concentration of the drug in the film than from the originally applied formulations after the evaporation of the solvent, leading to thermodynamic activity increase, which favors drug diffusion [24].

Nogueiras-Nieto et al. developed in situ gelling formulations of Poloxamer 407 and hydroxypropyl β-cyclodextrin (HP-*β*-CD), obtaining a polypseudorotaxane supramolecular structure that increases the delivery of antifungals (ciclopirox) and corticoid (triamcinolone) across human nails and hooves [25]. Later, the same group used methyl-β-cyclodextrin and Poloxamer 407 in a hydroalcoholic solution to deliver ciclopirox olamine and compared the results with other marketed ciclopirox olamine formulations. The proposed polypseudorotaxane lacquer increased the permeation and accumulation of the drug into the nail structure [22]. Chouhan et al. also demonstrated in vitro the enhancing effect of HP-*β*-CD for terbinafine ungual delivery [21]. Cutrin-Gomez et al., in 2018, showed the capacity of the soluble cyclodextrin derivatives (methyl-β-cyclodextrin and HP-*β*-CD) to modify the microporous characteristics of the nail plate via interaction with nail components. These modifications produce a significant increase in the drug permeability and drug accumulation in the nail [23].

There are a limited number of products on the market to treat nail alterations topically, based on hydroalcoholic solutions. Most of them are antimycotic drugs, formulated in a variety of nail lacquers bases. There are few nonmycotic agents to treat other nail alterations or protect from external aggressions.

Methyl sulfonyl methane (MSM), an important volatile component in the sulfur cycle, has long been thought of as a sulfur donor for sulfur-containing compounds such as methionine, cysteine, homocysteine, taurine, and many others [26,27,28]. MSM is present in keratin, where it is responsible for making nails hard and is a crucial component of collagen production [26]. MSM is structurally related to DMSO but differs in the oxidation state; it is a small (MW 94.14 Da) and hydrophilic compound (logP − 1.41). It is nonionizable in ambient conditions.

The presence of soft and brittle nails can also indicate a systemic deficiency of silicon [29,30]. Silicon also helps with the synthesis of glycosaminoglycans, along with collagen [30]. Barel et al. [31] evaluated the effect of the intake of supplements containing choline-stabilized orthosilicic acid (ch-OSA) on the skin, hair, and nails. After treatment, they observed a significant improvement in the fragility of nails and hair in the group using the ch-OSA. Dimethylsilanediol salicylate is an organic form of water-soluble silicon (MW 212.3 Da, log P 3.13, Log D (pH 5.5) 2.59). A salicylic radical could enhance properties for nail penetration.

Biotin has an important role in protein synthesis, especially in keratins, and has been claimed to contribute to healthy nails and hair. Oral biotin led to improved hardness and thickness in nails [6,32,33]. In vitro studies of keratinocytes showed the stimulation of cell differentiation and the production of cytokeratins [34]. They were also shown to stimulate lipid synthesis, responsible for the binding of keratinocytes in the nail plate [33]. Biotin is a small molecule of 244.3 Da, slightly soluble in water in its nonsalt form (logP 0.39, pKa 4.4) and leading to good permeability across nails.

We hypothesized that the topical administration of MSM, dimethylsilanediol salicylate, and biotin in polypseudorotaxane hydroalcoholic nail lacquer will effectively deliver these compounds across the nail barrier and thereby improve nail status and diminish signs of nail pathological alterations.

Hence, the aim of this study was to further investigate the transungual permeation/penetration profiles of MSM and biotin, as well as the amounts of sulfur and silicon in the nail, after the application of an experimental nail lacquer (Regenail^®^: REG) and a commercial nail lacquer (Betalfatrus^®^: BET). Secondary objectives included the evaluation of the clinical efficacy and safety of the experimental lacquer and of the user-friendliness, as gauged by the subject’s self-assessment and a medical examination and the effect in terms of the improvement of alterations in nails achieved by Regenail^®^.

## 2. Materials and Methods

### 2.1. Materials

MSM (10% *w*/*w*) and bamboo glycolic extract (Pracofar S.A., Barcelona, Spain), biotin (0.2% *w*/*w*) (Siemgluss Iberica S.A., Barcelona, Spain), HP-*β*-CD (8.7% *w*/*w*) (molar substitution degree of 0.65 and molecular weight of 1399 Da, HPB Kleptose, Roquette, Lestrem, France), dimethylsilanediol salicylate (0.246% *w*/*w*, Exsymol, Monaco), sodium lauryl sulfate (0.87% *w*/*w*, BASF, Barcelona, Spain), ethanol 96° (34.7%, Alcoholes Oliva, Barcelona, Spain), and purified water were used for the experimental nail lacquer (REG) production. Components were added one by one, from the least to the most concentrated, to the ethanol-water mixture.

The commercial nail lacquer (BET) chosen as a reference had the following composition: *Equisetum arvense*, methyl-sulfonyl-methane (~5.55% *w*/*w*), hydroxypropyl-chitosan, ethanol, water, and diethylenglycol monoethylether.

### 2.2. Methods

#### 2.2.1. Regenail Formulation Characterization

The produced formulation, Regenail, was characterized in terms of appearance (visual observation) and HPLC quantification of biotin and MSM. These determinations assessed the stability according to ICH long-term conditions 25 °C/60% HR, 30 °C/75% HR and in accelerated conditions 40 °C/75% HR. The HPLC quantification was as described in Section 2.2.2.

Finally, a release experiment was performed on Franz cells (VidraFoc, Barcelona, Spain; 100 mL volume and an effective diffusional area of 2 cm^2^) using a dialysis membrane (10–12 kDa). One milliliter of REG was placed in a donor compartment (*n* = 6) and 0.3 µL samples were taken at 1, 2, 4, 20, 22, and 24 h and replenished with the same volume of fresh receptor medium (5% HP-β-CD in phosphate buffered saline (PBS, pH 7.4, to maintain sink conditions). The system was continuously stirred at 500 rpm and maintained at 37 °C throughout the experiment. Biotin and MSM release were quantified according to the methods described in Section 2.2.2.

#### 2.2.2. In Vitro Penetration of the Nail Plate

We created in vitro permeation profiles of MSM and biotin (only in the case of REG), and recorded the amounts of sulfur and silicon in the nails after treatment with the experimental nail lacquer or the commercial nail lacquer.

##### Permeation Study

The bovine hooves were obtained from freshly slaughtered cattle, stripped of adhering cartilaginous and connective tissue. Hooves were cut into flat sections of approximately 300–700 µm in thickness and frozen until use.

The prepared hoof membrane was used in a Franz diffusion cell (*n* = 6 per formulation) with an effective diffusion area of 0.196 cm^2^. For this, hoof samples were cut into small discs using an 8-mm punch and placed between two PTFE adapters with an O-shaped ring of 5 mm in diameter. The ratio between the area of the O-shaped ring and the bovine hoof samples was 0.392 and, therefore, in accordance with Palliyil et al. [35], the lateral diffusion caused by the edge effect is not expected to have a significant influence on our results. The receptor compartment was filled with 5 mL of phosphate buffered saline (PBS, pH 7.4) and with 5% of HP-β-CD, and the cells were equilibrated at 37 °C in a water bath with magnetic stirring.

Two milliliters of the nail lacquers or PBS (blank) were placed in the donor compartment to avoid depletion during the 11 days of the experiment. Once a day, a 1 mL sample of receptor medium was sampled and replenished with the same volume of fresh medium. The receptor fluid was sampled for the determination of MSM by gas chromatography (GC) and biotin by HPLC. In order to determine the amounts of biotin, silicon and sulfur that remained in the hoof after the permeation experiment, hooves were cleaned with distilled water and dried using cellulose paper for further quantification.

##### Biotin Quantification in Receptor Medium and Hooves

The biotin content of the samples (from receptor medium, *n* = 6; from hoof, *n* = 6) was directly analyzed by a validated HPLC (Water Corporation, Barcelona, Spain) method (mobile phase: Acetonitrile/buffer (1 g sodium perchlorate, 10 mL phosphoric acid up to 1 L of purified water) (10:90), flow 1.0 mL/min, column Symmetry 200 C18 3.5 µm, 150 mm × 4.6 mm). Biotin was extracted from hoof samples by incubation with 5 mL of 5% methanol in a PBS solution for five days (at 25 °C). To quantify biotin content, a calibration line in the range 0.7–506 µg/mL was prepared. The cumulative amounts permeated were normalized versus the permeation surface. The slope of cumulative amounts (normalized by surface) vs. time (h) was calculated.

##### MSM Quantification in Receptor Medium

The MSM content of the receptor medium samples (*n* = 6 per formulation) was analyzed by CG (Agilent Technologies, Barcelona, Spain; mobile phase helium, flow 8.0 mL/min, column DB-1 50 m, 0.53 mm internal diameter, 5 µm). A calibration curve in the range of 3 to 13,300 µg/mL was prepared in a methanol solvent. The cumulative amounts released were normalized versus the permeation surface.

##### Sulfur and Silicon Quantification in Hooves after Permeation

In order to determine the amount of sulfur and silicon in the hooves, they were treated by digestion for 120 min with 2 mL of a solution of 2.5% TMAH (tetramethylammonium hydroxide, Sigma-Aldrich, Barcelona, Spain) in water at 90 °C. The intensity value obtained for the blank was subtracted from the values obtained with each one of the samples. The sulfur and silicon content in hooves was analyzed by ICP-OES (Inductively Coupled Plasma-Optical Emission Spectrometry). Sulfur was determined at 182.0 nm and silicon was determined at 288.2 nm.

#### 2.2.3. In vitro Cytotoxicity Assay

The cytotoxic effect of MSM was evaluated in vitro (*n* = 6) by direct contact with the cell, following the ISO 10993-5:2009 recommendation guidelines [36].

The HaCaT cells were seeded in a 96-well plate at a density of 10,000 cells/well, in a fresh culture medium supplemented with 10% fetal bovine serum, penicillin (100 IU/mL), and streptomycin (100 μg/mL) (Fisher Scientific, Barcelona, Spain). The plates were incubated for 24 h in a humidified atmosphere of 5% CO_2_ at 37 °C and treated with different concentrations of MSM from 10 µM to 0.003 µM, diluted in growth medium. For cell proliferation quantification, the general cell viability endpoint MTT (Sigma-Aldrich, Barcelona, Spain) reduction (3-(4,5-dimethyl-2-thiazolyl)-2,5-diphenyl-2H-tetrazolium bromide) was used [37]. Accordingly, the previous culture medium was removed and replaced with a fresh medium containing MTT (1:10). The cells were incubated at 37 °C for 3 h; after this time, the medium was removed and the intracellular formazan crystals were solubilized and extracted with dimethylsulfoxide (Panreac, Barcelona, Spain). After 15 min at room temperature, the absorbance was measured at 550 nm in a microplate reader (Perkin Elmer, Barcelona, Spain).

#### 2.2.4. TNFα and IL8 RNA Isolation and RT-PCR

RNA was isolated from cell samples (*n* = 6) using the method described by Chomczynski [38], using the RNeasy kit (Qiagen) and treating with DNAse-I to remove any contamination from genomic DNA.

The purity of the RNA was estimated by 1.5% agarose gel electrophoresis, and the RNA concentration was determined by nano-drop spectrophotometry (Thermo Fisher Scientific, Barcelona, Spain).

Reverse transcription of 1 μg RNA for complementary DNA (cDNA) synthesis was performed using a First-strand Synthesis kit (Takara-Clontech, Saint-Germain-en-Laye, France). This cDNA from treated or untreated cells (control) was used to determine the relative gene expression of TNFα and IL-8 through RT-qPCR.

Real-time (RT)-qPCR was performed in a QuantStudio 5, Applied BioSystem (Barcelona, Spain) apparatus. For the normalization of cDNA loading in the PCR reaction, the amplification of ACT for every sample was used. Relative changes in gene expression were calculated using the Pfaffl method, following standard procedures [39].

#### 2.2.5. Clinical Study Design

The main objective of the study was to evaluate the clinical efficacy and subjective perception of the improvement in nail alterations achieved by the experimental nail lacquer (REG) or the commercial nail lacquer (BET). The safety of both products was also evaluated as a secondary endpoint.

Thirty volunteers (between 18 and 65 years old) from both sexes were recruited to participate in the study. The inclusion criteria were: Both genders, general good health (physical and psychological), alterations on the surface of the nails of both hands, adequate understanding of the clinical study, agreement to participate, and availability to attend the visits. No other topical nail product could be used during the study and any other treatment that could affect the evaluation of the study endpoints was forbidden. Each subject received oral and written information concerning the studied product. Written consent was obtained before any study-specific procedures, in accordance with the Helsinki Declaration.

Eligible subjects were randomly assigned to an experimental group. Patients were treated for a period of 28 days. Both products were applied once daily, preferably at night (to ensure a contact time of 6 h), with a brush, covering the complete (cleaned) nail. After application of the nail lacquer, patients were instructed not to clean their hands, because the obtained film in nails is hydrophilic. The following day, patients could do their routine daily cleaning (shower, hand washing, etc.). Nails’ evolution was evaluated at day 28, compared with D 0 (the baseline). On day 14, volunteers came to the study site for a control visit.

On day 0 and day 28, the nails were examined by a dermatologist to evaluate the nail alterations’ progression (Beau’s lines and onychorrhexis). A categorical scale ranging from 0 (alteration absence) to 4 (severe alteration) was used by the dermatologist for evaluation. The nail roughness was evaluated with PRIMOS-CR^®^ (Canfield Scientific, NY, USA). The transonychial water loss (TOWL) was also evaluated with the Tewameter^®^ TM 300 (Courage + Khazaka electronic GmbH, Köln Germany). Participants had washed their hands prior to the topographical evaluation. Measurements of camera distance and ambient illumination were standardized.

A subjective questionnaire was included at the end of the study to evaluate product usability and efficacy. A Likert scale (from 0—totally disagree, to 5—totally agree) was employed for this evaluation.

The compatibility of the product with nails and any undesirable effects were assessed at every visit by a dermatologist.

#### 2.2.6. Data Analysis

The slope of the linear portion of the cumulative amounts vs. the time of permeation profile was estimated as the pseudo steady-state flux (Js) of penetrant permeation. The lag times (Tlag) for each active compound were derived from the x-intercept of the slope at pseudo steady-state. The permeability coefficients (Kp) of each active compound were calculated as compound flux/compound concentration in the donor chamber. The diffusion parameter P_1_ and the portioning parameter P_2_ were calculated according to Equations (1) and (2), respectively. The results were expressed as mean ± standard deviation (SD) and were statistically analyzed with Minitab software (Coventry, United Kingdom).

Kp = P_1_·P_2_ (1)

Tlag = 1/(6 P_2_) (2)

The significance of the effect of the treatment over time on the response variables was evaluated using an ANOVA test. A significance value of 0.05 was established for all statistical tests used in the data analysis.

## 3. Results

### 3.1. Formulation Characterization

**Figure 1:**
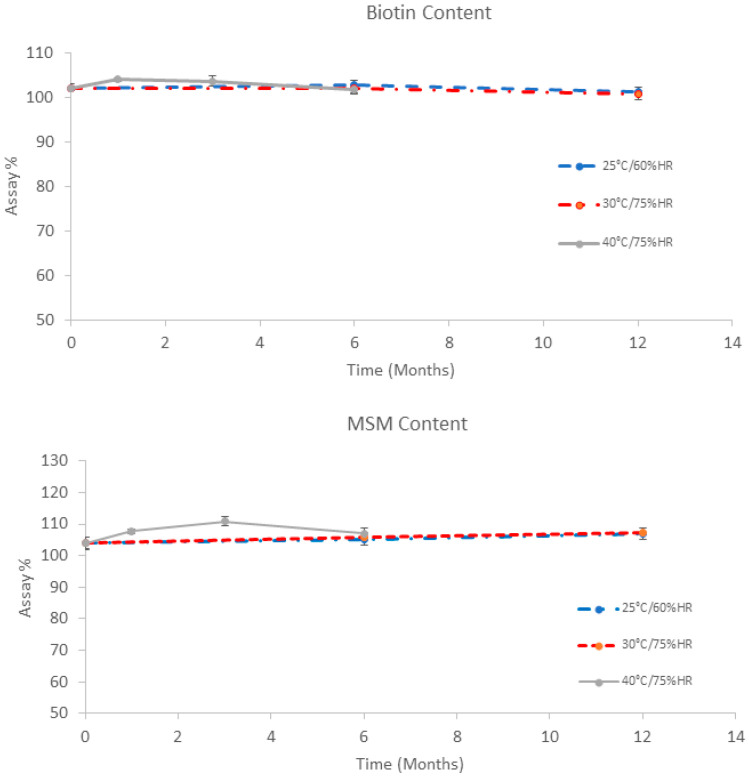
Biotin (**upper panel**) and methyl sulphonyl methane (MSM) (**lower panel**) content of Regenail^®^ (REG) in stability studies under different climate conditions.

Figure 1 shows the results of biotin and MSM under different climatic conditions for 12 months. The data show low variation (less than 5%) compared with the initial time point for both active compounds in all conditions. The results at 30 °C/75% HR almost overlap with those at 25 °C/60% HR for both MSM and biotin, with no differences shown between these stability conditions. According to ICH Q1E [40], if no significant variation (less than 5%) is seen in the accelerated stability conditions, then an extrapolation of long-term conditions up to two years could be stated, based on the obtained results. A long-term study will continue until the proposed extrapolated shelf-life (two years) to confirm the obtained results. The regression equations at 25 °C/60% HR (long-term conditions at climate zone II) were for biotin y = 102.57 − 0.067*Time and for MSM y = 103.9 + 0.25*Time. The regression equations at 30 °C/75% HR (long-term conditions at climate zone IVb) were for biotin y = 102.43 − 0.117*Time and for MSM y = 104.02 + 0.275*Time. To evaluate whether there are differences in the regression lines, ANCOVA analysis (covariance analysis) was performed. In both biotin and MSM, there were no differences in interaction factor Time x Climate condition (*p* < 0.05), which means that the regression line slopes are considered parallel and the factor climate conditions (which represents the *y*-intercept) is not significant (*p* < 0.05). The overall biotin and MSM regression lines could be considered coincident at both climate conditions (25 °C/60% HR and 30 °C/75%HR).

**Figure 2:**
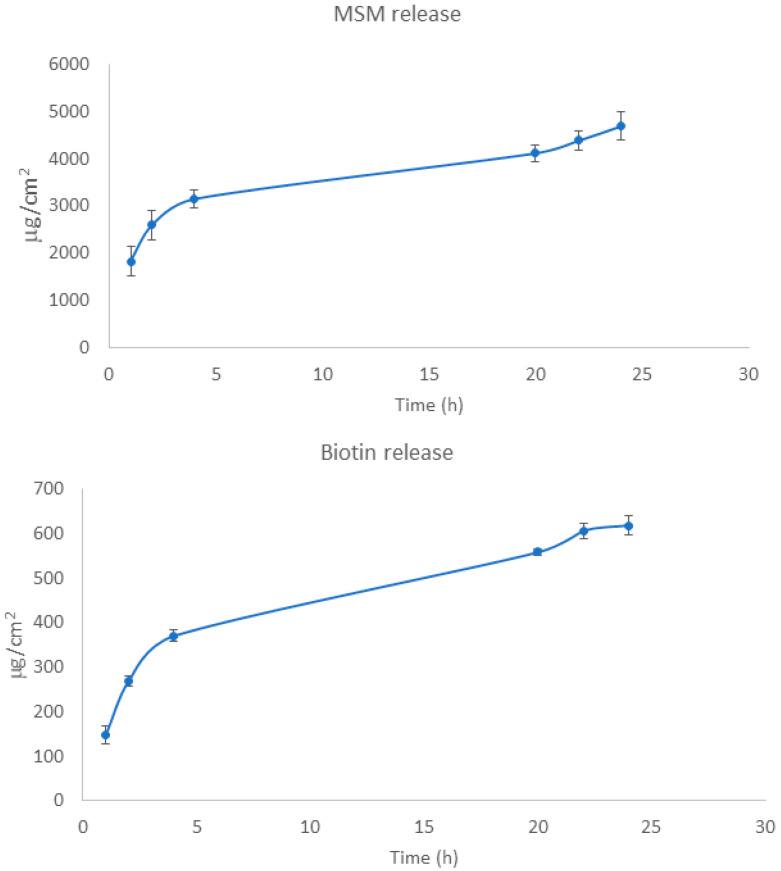
MSM (**upper panel**) and biotin (**lower panel**) release over time from REG nail lacquer.

Release experiments (Figure 2) showed a slow release of both compounds, probably due to the dialysis membrane being the rate-limiting step (the active compounds are not expected to be encapsulated, because the high solubility in the hydroalcoholic vehicle). This membrane was chosen to prevent the HP-β-CD polypseudorotaxane complex from passing through to the receptor medium. In addition, the small pore of these membranes could be more similar to hoof pores than the standard membranes of 0.22 or 0.45 µm usually employed in release experiments. A limitation of release experiments is that artificial membranes, such as cellulose dialysis membranes, are not sensitive to permeation enhancers. For this reason, in vitro permeation with hooves was performed.

### 3.2. In Vitro Permeation Study

**Figure 3:**
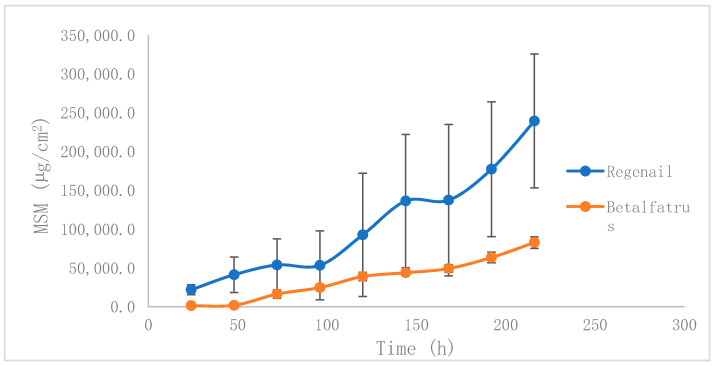
Permeation profiles of MSM through the bovine hoof plate as a model of human nail permeation.

Nail penetration of active compounds was investigated in Franz diffusion cells after topical application of experimental nail lacquer (REG) or commercial nail lacquer (BET) to hooves for 11 days. Infinite dose conditions were set up to assess formulation differences in terms of the permeation profiles. Both tested products permeated the bovine hoof plate, but differences in permeation parameters were noticed, related to the active compounds and the formulation. The permeation profiles of MSM across the hoof are shown in Figure 3. The transungual permeation parameters of active compounds containing the formulations are presented in Table 1.

The calculated permeation steady state flux of MSM through REG formulation, across the hoof plate, was observed to be 10,839.695 μg/h/cm^2^, more than double that of the reference product. The lag times (Tlag) were statistically different: 105.12 h for REG vs. 98.77 h for BET (*p* < 0.05). The mean values for the P_1_ (diffusion-related parameter) and permeability coefficients (Kp) of MSM were also more than double the corresponding values for the reference.

However, P_2_ (the partitioning-related parameter) was similar for the two nail lacquers, thus indicating that the partitioning coefficient of the MSM between the formulation and the nail is similar, so both formulations are similar in terms of the relative polarity of the MSM.

These results clearly indicate that REG allows higher permeation levels of MSM than those obtained with the reference product BET.

Additionally, these data were normalized by the amount of MSM applied in each test (the MSM concentration between both products is different: 10% *w*/*w* for REG and around 5.5% *w*/*w* for BET).

When these normalized values are considered, the profiles of both formulations get closer. The mean values for the P_1_, P_2_, and Kp of MSM that permeated through hooves showed negligible differences between the two formulations. The permeation steady state flux values, however, were significantly different: 1083.97 μg/h/cm^2^ for REG vs. 647.89 μg/h/cm^2^ for BET. Although most of the differences between permeation parameters were caused by different MSM concentrations, the increased flux after dose normalization indicates the superiority of the test vehicle over the commercial formulation. According to the molecular structure of MSM, it is not expanded to encapsulate or interact with HP-β-CD, so the increase in transungual flux could be caused by the shelf promoting effect of the formulation. It was demonstrated previously [22,23,25] that cyclodextrin-soluble polypseudorotaxanes increase the size of nail channels and interact with keratin residues, reducing protein folding. Sodium lauryl sulfate could also increase the diffusivity of MSM by contribution to protein interaction. MSM is not expected to reduce disulfide bonds. In addition, the proposed formulation can increase the absorption of hydrophilic active compounds despite the well-known solubilization capacity of lipophilic molecules, which is the main promotion mechanism in these compounds.

When permeated concentrations of MSM at 24 h (around 21,700 µg/cm^2^) are compared with release concentrations at the same time point (around 1800 µg/cm^2^), we see the enhancing effect of REG caused by the interaction of the system with the hoof structure, compared with the pure diffusion seen in release experiments across dialysis membranes.

The amount of sulfur obtained through the MSM at the end of experiments after application of REG formulation was 948.66 ppm, 2.3-fold higher than that obtained with the BET solution (412.78 ppm).

**Figure 4:**
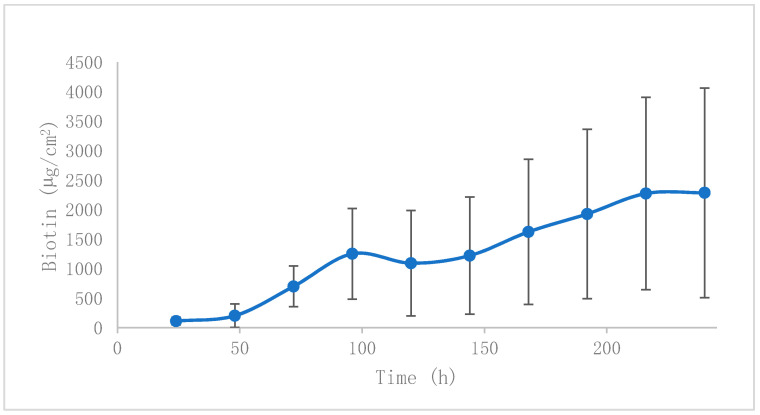
Permeation of biotin across the bovine hoof plate.

Biotin profiles from REG could not be compared with the reference product because it does not contain biotin. The permeation profiles of biotin across a hoof are shown in Figure 4. The transungual biotin parameters are shown in Table 1. As P_1_ is higher than P_2_, diffusion is the main driving force that allows for active penetration. There are no previous investigations that have described the topical absorption of biotin, so this is the first time that transungual delivery of biotin has been described. It is shown that this route of administration could lead to topical absorption across the nail plate, leading to pharmacological action at the nail bed.

Biotin is also a hydrophilic molecule, and is not expected to be encapsulated, but it could form complexes with HP-β-CD [41], to ease biotin permeation, together with the previously described enhancement mechanism of CD-polypseudorotaxanes.

Although hooves are a good model to study transungual permeation, it should be considered that hooves have some differences compared to human nails. They are more porous and permeable than human nails due to the less dense keratin network. In addition, disulfide bonds are less abundant because of the lower content of cysteine, so hooves are less affected by enhancers that reduce these bonds [4]. Nail diseases, like psoriasis and onychomycosis, lead to higher porosity than in healthy nails; in this case, hoof results could be extrapolated more accurately [22]. Nevertheless, the study had comparative purposes, and was not used to obtain human absolute values. The experiment was performed according to the infinite dose paradigm, in other to maximize formulation differences and avoid analytical method quantification issues. In clinical applications, a lower dose of the nail lacquer is applied, which is another limitation of the permeation study.

When permeated concentrations of biotin at 24 h (around 90 µg/cm^2^) are compared with release concentrations at the same time point (around 140 µg/cm^2^), we observed a small reduction in hoof absorption compared with pure diffusion. This could be caused by a possible interaction between biotin and HP-β-CD [41], or even an interaction of the amino and/or carboxylic groups of the biotin molecule with hoof keratins.

Similar to MSM, the silicon content was determined in the reference product; the values obtained for silicon were around 150 ppm, while this content in the test product is around 300 ppm. The silicon values in the bovine hoof plate after diffusion studies were 3.54 ± 1.81 and 1.33 ± 0.77 ppm for the new nail lacquer and the reference, respectively. Although there are no statistical differences between treatments, the higher percentage of active compounds remaining in the membranes at the end of the permeation studies in REG is probably due to the higher silicon content of the new product and/or the nature of the silicon (dimethylsilanediol salicylate versus silicon in *Equisetum arvense*), as described in Section 2.2. In addition, salicylate could act as a permeation enhancer [4] and could form a complex with HP-β-CD by the aromatic ring [42], increasing the diffusion of the active ingredient.

The tissue levels of silicium decrease with ageing [43]. All silanols are known to prevent the formation of advanced glycation end products (AGEs), to have antioxidant properties, to stabilize the structure of the extracellular matrix, and to induce the production of collagen. In addition, dimethylsilanediol salicylate also has anti-inflammatory and antioxidant properties and stimulates heat shock protein expression [44,45]. Brittle nails are often improved by silicon uptake [46]. The bioavailability of organic silicium (dimethylsilanediol salicylate) allows it to quickly integrate into the structure of the nails and could compensate for its natural age-related loss.

### 3.3. Cytotoxic Effect of MSM

**Figure 5:**
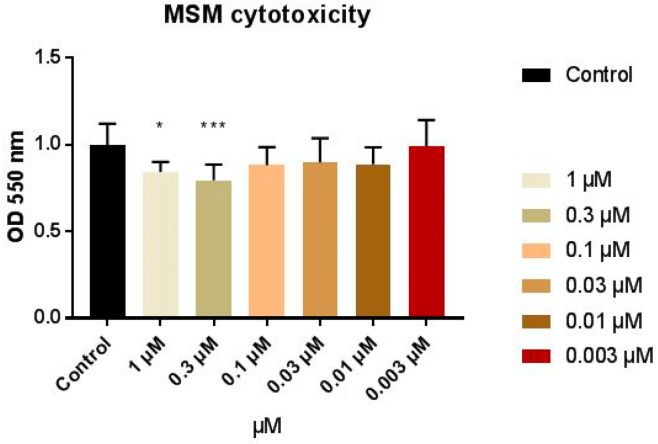
HaCaT cell viability by MTT after proliferation under different concentrations of MSM. (*) shows statistical differences at a significance level of α = 0.10. (***) shows statistical differences at a significance level of α = 0.05.

First, the cytotoxic effects of a wide concentration range of MSM (0.003–1µM) on human keratinocytes (HaCaT line) without stimulation were assessed with a MTT test. Compared to the control (cells cultured in the absence of MSM), no differences in cell viability or proliferation were detected at doses of 0.1, 0.03, 0.01, or 0.003 µM of MSM. However, significant reductions (*p* < 0.05) in cell viability occurred at 0.3 µM of MSM (Figure 5). Concentrations of 1 µM presented a significant reduction when considering a significance level α = 10%, but not at 5%. When different concentrations were compared, there was no statistically significant difference, showing that the slight reduction in cell viability is due to experimental variability. The significance obtained at 0.3 µM compared with the control could be considered a statistical artifact.

MSM is widely used orally for arthritis symptom relief due to its anti-inflammatory and antioxidant properties, with a broad dose range (up to 1.5 g/day) [47] with a good safety profile. In addition, it was considered to be a generally recognized as safe (GRAS) ingredient by the FDA (United States Food and Drug Administration) up to 4845.6 mg/day [27]. This fact is in concordance with the obtained results, despite the statistical artifact.

### 3.4. Anti-Inflammatory Effect of MSM

In order to assess the anti-inflammatory activities of MSM, human keratinocyte (HaCaT line) cells were exposed to LPS (100 ng/mL) in the presence or absence of MSM, and the gene expression of TNF-α and IL-8 were measured. Because of LPS exposure, the TNF-α and IL-8 gene expression significantly increased by 848.2 ± 69.8% and 710.4 ± 83.2%, respectively. MSM treatment prevented significant increases in TNF-α and IL-8. The results indicate that MSM at 0.1 and 1 µM significantly decreased TNFα expression by 76.4 ± 8.6% and 81.9 ± 7.4%, and IL-8 expression by 66.1 ± 11.9% and 62.7 ± 10.3%, respectively, compared to the Control + LPS.

**Figure 6:**
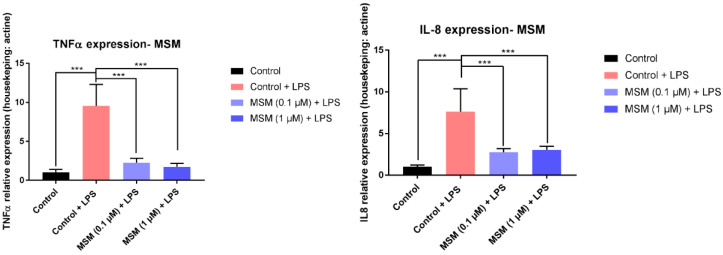
Effect of MSM on gene expression of TNF-α and IL-8 of LPS activated human keratinocytes (HaCaT line). (***) show statistical differences at a significance level of α = 0.05.

It was found that MSM strongly inhibits IL-8 and TNF-a production in LPS-stimulated human keratinocytes (Figure 6). Our data are in accordance with previous publications. So, in bone marrow-derived macrophages, MSM inhibits ROS production and attenuates the transcriptional expression of IL-1a, IL-1b, IL-6, and NLRP3 [48]. Furthermore, Kim and colleagues demonstrated that MSM blocks NF-kB activation, thereby inhibiting NF-kB-mediated transcription of inflammatory genes [49]. Previous findings demonstrate an increased expression of tumor necrosis factor (TNF)-α, nuclear factor-kappa B, IL-6, and IL-8 in psoriasis-affected nails [50]. An important observation of our study is that treatment with MSM reduced the expression of (TNF)-α and IL-8; like a principal component of REG, the proposed nail lacquer could be used not only for the treatment of nail alterations, but also for the treatment of nail diseases related to inflammation, such as nail psoriasis. On the other hand, MSM enhanced the passage of other drugs such as ciprofloxacin across the porcine membrane, as compared to not using MSM [51]. This could be useful to increase the therapeutic effect of antimicrobial drugs in the nail plate.

### 3.5. Clinical Evaluation

The main nail brittleness alterations found in the volunteers were Beau’s lines and onychorrhexis. Beau’s lines are transversal depressions caused by temporary nail bed mitosis disruption and can be associated with everything from trauma to rheumatic disease, malaria, pemphigus, and Raynaud’s disease, among other pathologies [52]. Beau’s lines-related conditions could be improved with adequate nail hydration and compounds that increase fibroblast proliferation [53]. Onychorrhexis is commonly observed in advanced age, resulting in brittle nails, but also appeared in a more severe pattern in collagen vascular disease, rheumatoid arthritis, psoriasis, and nutritional deficiencies [54].

**Figure 7:**
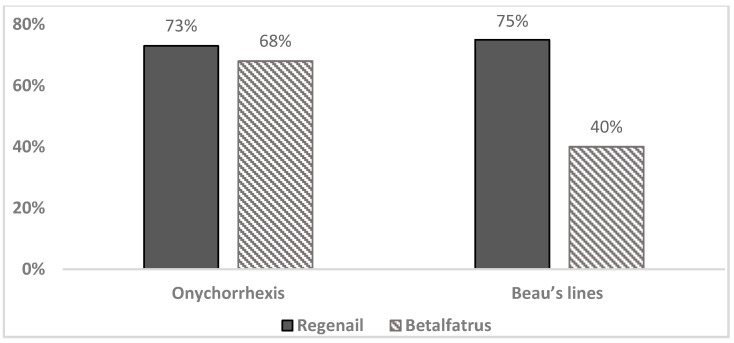
Percentage of volunteers whose nail onychorrhexis and Beau’s lines improved after 28 days of treatment versus the baseline (%).

According to the dermatologist’s clinical evaluation, the onychorrhexis decreased in the BET group, with an improvement observed in 68% of subjects after 28 days of application. As shown in Figure 7, the percentage of volunteers with improvement of onychorrhexis was 73% in the REG group after 28 days of treatment. The observed difference was comparable between the two treatment groups.

Improvements in Beau’s lines were observed by a dermatologist in both treatment groups (Figure 5). In the REG group, the severity of the Beau’s lines decreased in 75% of subjects after 28 days of treatment compared with the baseline. In 40% of the subjects who applied BET, the Beau’s lines severity decreased after 28 days of treatment compared with the baseline.

As previously described, Beau’s lines and onychorrhexis are indirect markers because they could appear in different nail alterations, such as onychomycosis and nail psoriasis. Regenail’s properties could be useful in both pathologies. MSM has been demonstrated to have anti-inflammatory activity, reducing some of the cytokines overexpressed in psoriasis. It was previously shown that MSM has a high permeation rate and could be available in the nail matrix. It is difficult to correlate the levels of MSM found in the receptor compartment of Franz cells (Section 3.2) with the in vitro concentrations assayed in cell experiments (Section 3.3 and Section 3.4) because of the different experimental protocols and scale (tissue vs. cell), but the clinical study showed that the proposed treatment has benefits over the reference product: It improved signs of nail alterations, and delivered safe and effective concentrations of active ingredients to human nails.

Cyclodextrins exhibit antimicrobial properties because they could interact with the protein and lipid components of a microorganism [42]—for example, ergosterol of the fungal membrane, increasing permeability and the loss of cell content. This could have additional benefits for onychomycosis treatment.

Biotin could act as a promoter of keratin production to restore nail function after disease, and the silicon derivate helps to reduce the nail fragility caused by the disease. Chessa et al. reported that nail brittleness could be improved by correct nail hydration, protection from external aggressions (i.e., detergents, irritants), biotin supplementation (to increase hardness), and intake of silicon as well as other trace elements [55].

**Figure 8:**
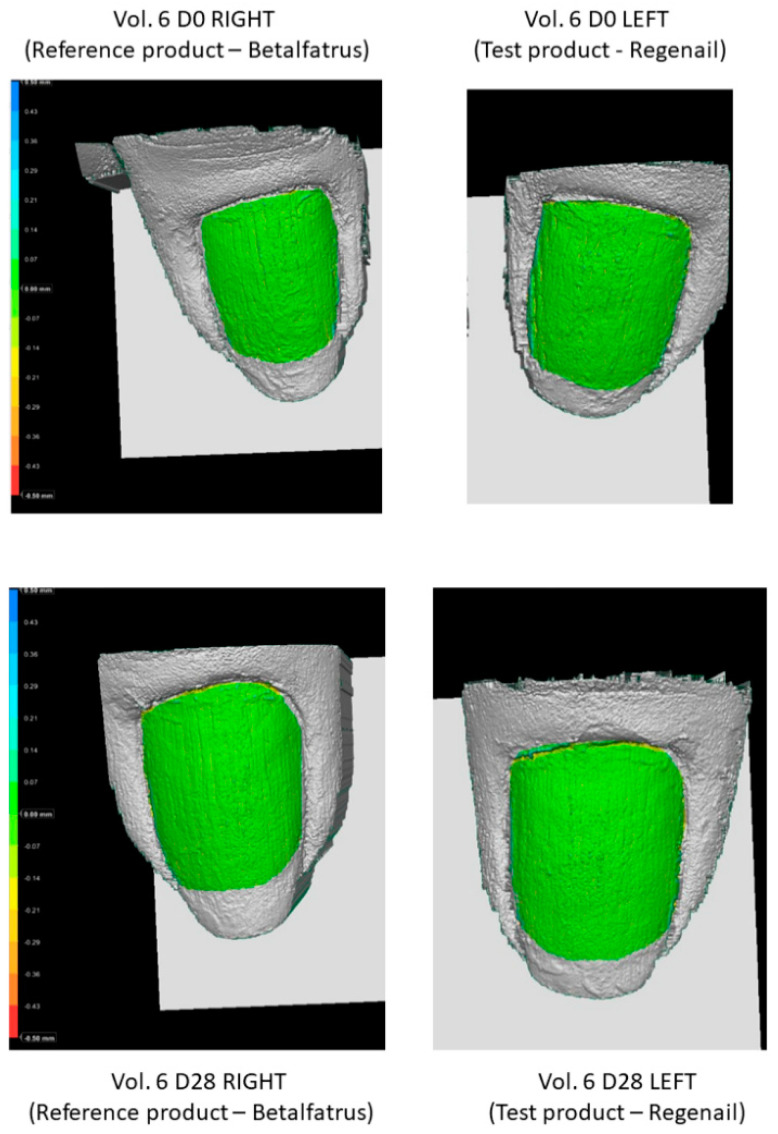
Nail images after the application of Betalfatrus and Regenail.

PRIMOS-CR is a high-resolution small-field 3D system used to evaluate the surface characteristics of tissues in vivo. It allows us to quantify the topography of the skin or other body surface with the parameter Sa or arithmetic mean of surface roughness. In addition, software could produce color height maps (Figure 8). The evaluation showed a decrease in surface roughness (Sa)—that is, an anti-roughness effect—after REG administration. Sa decreased by 12% versus the baseline after 28 days of REG application (day 0: 0.69 ± 0.38; day 28: 0.61 ± 0.32). The evaluation of nail roughness in the group of volunteers after the application of BET showed a decrease of only 1% after 28 days of application (day 0: 0.66 ± 0.45; day 28: 0.65 ± 0.37). However, there was no significant difference in the surface roughness between treatments.

Alterations of nail topography (roughness) could be derived from ridges (Beau’s lines and onychorrhexis) but also nail pitting (Rosenau’s depressions), produced by Alopecia areata, lichen, pemphigus, and psoriasis [13]. Regenail has been demonstrated to reduce roughness and improve signs of possible nail diseases.

A decrease in TOWL was observed after the application of REG and BET, but without significant differences. So, after 28 days of application of REG, the TOWL decreased by 6%. In the case of BET, the same parameter was decreased by 4% after 28 days of application. There was no significant difference in nail permeability between the treatments. The lack of TOWL changes in both formulations reflects a lack of structural nail alterations and the maintenance of physiological performance and integrity.

Other studies have also shown improvements in nail condition with topical formulations. For example, in one study, 10% urea lacquer was applied once or twice daily for 28 days. The study demonstrated significant improvements in nail surface morphology and nail plate consistency with the urea lacquer [56]. Previous clinical studies showed that hydroxypropyl-chitosan nail lacquer, when applied to fingernails, reduced longitudinal grooves, lamellar splitting, and nail fragility in women with nail plate alterations [57].

Our results ae in accordance with those of other studies in which the authors were able to demonstrate that HP-*β*-CD enhanced the permeation of other compounds across the nail plate by virtue of increasing the nail hydration ability as well as the aqueous solubility of the drug without damaging the nail plate integrity. This establishes the suitability of HP-*β*-CD as a nail-friendly transungual permeation enhancer for poorly water-soluble drugs [21]. Additionally, the results are in accordance with those of Cutrin-Gomez et al., whose in vivo study optimized a ciclopirox olamine lacquer based on similar cyclodextrin polypseudorotaxanes that was applied to the nails of healthy volunteers for 45 days, leading to no negative effects on the nail surface [58].

### 3.6. Self-Evaluation Questionnaire

**Figure 9:**
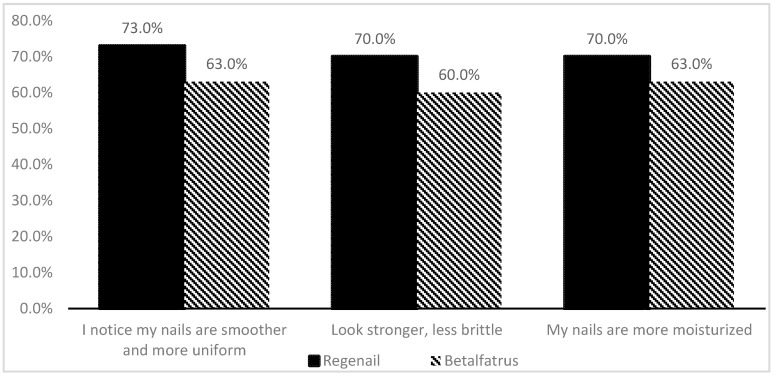
Subject assessment of the efficacy (by % of satisfied volunteers) of Regenail and Betalfatrus.

Although the majority of subjects in this study considered fingernail resistance, smoothness, glossiness, growth, and general condition to be improved across all treatments, it can be seen that subjects rated REG higher than BET in terms of nail brittleness, nail smoothness, and moisturizing ability (Figure 9).

### 3.7. Safety Evaluation

None of the patients that started the study had any adverse effect (AE) during the study period, either pertaining to the treated area or system-wide. As no serious or treatment-related AEs leading to temporary or definitive discontinuation of the study were reported, it is possible to confirm that the treatments were well tolerated and demonstrated very good cutaneous compatibility. There are no particular safety concerns.

With the nail lacquer presented in this research, we demonstrated the good permeation profile of biotin with the proposed cyclodextrin polypseudorotaxanes vehicle. The water content of the product (around 35%) and the moisturizing effect of cyclodextrin led to adequate hydration of the nail. The higher water content of the liquid formulations is likely to hydrate the nail plate, generating more microporous channels for the permeant and therefore leading to an increased permeation of active ingredients. The active ingredients, MSM, biotin, and dimethylsilanediol salicylate, make the product a good candidate to treat nail alterations derived from ungual disease, such as psoriasis and onychomycoses. Further clinical studies should be performed to corroborate this hypothesis.

## 4. Conclusions

A new cyclodextrin polypseudorotaxanes nail lacquer, containing biotin, MSM, and dimethylsilanediol salicylate, was studied in vitro and in vivo. An improved transungual permeation profile was exhibited for both MSM and biotin, and increased amounts of sulfur and silicon were observed in the nail matrix after the studies. MSM demonstrated good anti-inflammatory activity in human keratinocytes, making it a promising product to treat nail inflammatory disorders. Clinical studies showed a good safety profile and efficacy on nail alterations, such as Beau’s lines, with a smoothening effect on the ungual surface and improved moisturizing and hardness.

## Figures and Tables

**Table 1:** Transungual permeation parameters of test and reference formulation. (*) = statistical differences between formulations with a *p*-value < 0.05.

Formulation	Active Compounds	Js (μg/h/cm^2^)	Kp (cm/h)	P_1_ (cm)	P_2_ (h^−1^)	T_lag_ (h)	Active Content (µg/mg)
Regenail	Biotin	74.98 ± 35.34	0.0187 ± 0.0102	6.7163 ± 4.5610	0.00279 ± 0.00118	59.72 ± 5.34	0.161 ± 0.098
	MSM	10,839.695 * ± 5786.239	0.054 * ± 0.014	34.185 * ± 8.661	0.00159 ± 0.00098	105.12 * ± 3.56	--
Betalfatrus	MSM	3563.382 * ± 963.993	0.0324 * ± 0.011	19.198 * ± 4.821	0.00170 ± 0.00125	98.77 * ± 3.01	--
